# A Rare Case of Syphilitic Charcot Arthropathy Involving the Foot in an Asian Patient: A Case Report and Review of Literature

**DOI:** 10.7759/cureus.101901

**Published:** 2026-01-20

**Authors:** Teddy Cheong, Kinjal Mehta

**Affiliations:** 1 Orthopaedic Surgery, Changi General Hospital, Singapore, SGP

**Keywords:** asian, charcot arthropathy, foot, syphilis, tabetic arthropathy

## Abstract

Syphilis is an infection caused by *Treponema pallidum*. Without treatment, neurosyphilis may develop and lead to complications such as Charcot arthropathy (CA). First described by Jean-Martin Charcot, this condition was initially recognized as a manifestation of syphilitic infection. With the advent of antibiotics, the incidence of syphilis declined; however, it has since re-emerged in regions including Europe, North America, and China. In recent years, there has been a relative paucity of literature on syphilitic CA, with most reports involving the hip or knee. This article describes a rare presentation of syphilitic CA affecting the foot and aims to contribute to the limited body of literature while underscoring the importance of considering syphilis as a differential cause of CA of the foot in the modern era.

A 37-year-old patient with a remote history of syphilis (diagnosed approximately 10-15 years earlier) presented with an atraumatic, progressively worsening swelling of the right foot over three months, associated with a progressive deformity of unknown duration. Imaging demonstrated features of CA involving the foot, with no radiological evidence of infection. A workup for potential etiologies was performed. Cerebrospinal fluid (CSF) analysis revealed a Venereal Disease Research Laboratory (VDRL) titer of 1:8 and a positive Line Immunoassay (LIA) syphilis CSF immunoglobulin G (IgG) test. The patient received intravenous benzylpenicillin therapy followed by deformity-correction surgeries. Four years after the initial operation, the patient was ambulating independently without assistive devices and had no wound complications.

Syphilitic CA is a serious complication and should be considered in the differential diagnosis of patients presenting with features of neuropathic arthropathy, even in the modern era. This is particularly relevant for cases involving the foot, where diabetes is the most common aetiology and may lead clinicians to erroneously assume a diagnosis of diabetic CA. Early consideration and confirmation of syphilitic CA enables the timely initiation of antibiotic therapy for tertiary syphilis, while the CA can be managed through deformity-correction surgery if required.

## Introduction

Syphilis is an infection caused by *Treponema pallidum* and can occur in several stages such as primary, secondary, and tertiary. In the absence of treatment, neurosyphilis can develop and result in complications such as Charcot arthropathy (CA) [1]. CA is characterized by progressive bony destruction with impaired nociceptive and proprioceptive innervation of the affected joints [2]. In 1868, Jean-Martin Charcot originally described this disease as a complication of syphilitic infection [3]. As antibiotics became more available, the incidence of syphilis decreased [4]. However, syphilis has re-emerged in regions such as Europe, North America, and China mainly due to travel and high-risk sexual activity [5,6]. Currently, diabetes is the most common cause of CA, and it most frequently affects the foot and ankle [7]. In recent years, there has been a relative paucity of literature on syphilitic CA, with most articles reporting on the involvement of hip, knee, and ankle joints [8-17]. This article presents a case of syphilitic CA involving the foot in an Asian patient and describes the associated long-term outcome. In addition, a review of the literature on recent case reports of syphilitic CA is presented.

This case report highlights the importance of considering syphilitic CA as a differential diagnosis in patients presenting with joint destruction consistent with neuropathic arthropathy involving the foot, even in the modern era. Through early consideration and confirmation of the diagnosis, important interventions to treat the underlying cause of CA can be performed. Informed consent was obtained from the patient for participation and publication purposes of this article. This article was written in accordance with the research guidelines determined by the local ethics review board.

## Case presentation

This article includes a 37-year-old patient of Asian descent with a medical history of bipolar disorder and syphilis, which was diagnosed approximately 10-15 years prior to the current presentation. The patient had no history of diabetes. In 2019, the patient presented to the emergency department for worsening right foot dorsal swelling of three months' duration. This was associated with a progressive deformity of the right foot of an unspecified duration, which started to affect the patient’s shoe wear and ambulation. There was no history of trauma, infection, open wounds, or complaints of pain in any other joints in the body. The patient was afebrile with stable vital readings. On physical examination, there was generalized swelling of the right foot with midfoot collapse and reduced gross sensation. Peripheral pulses were intact, and there were no open wounds. The ankle joint had a full, pain-free range of motion, and there were no signs suggestive of infection such as open wounds, warmth, or erythema.

**Figure 1:**
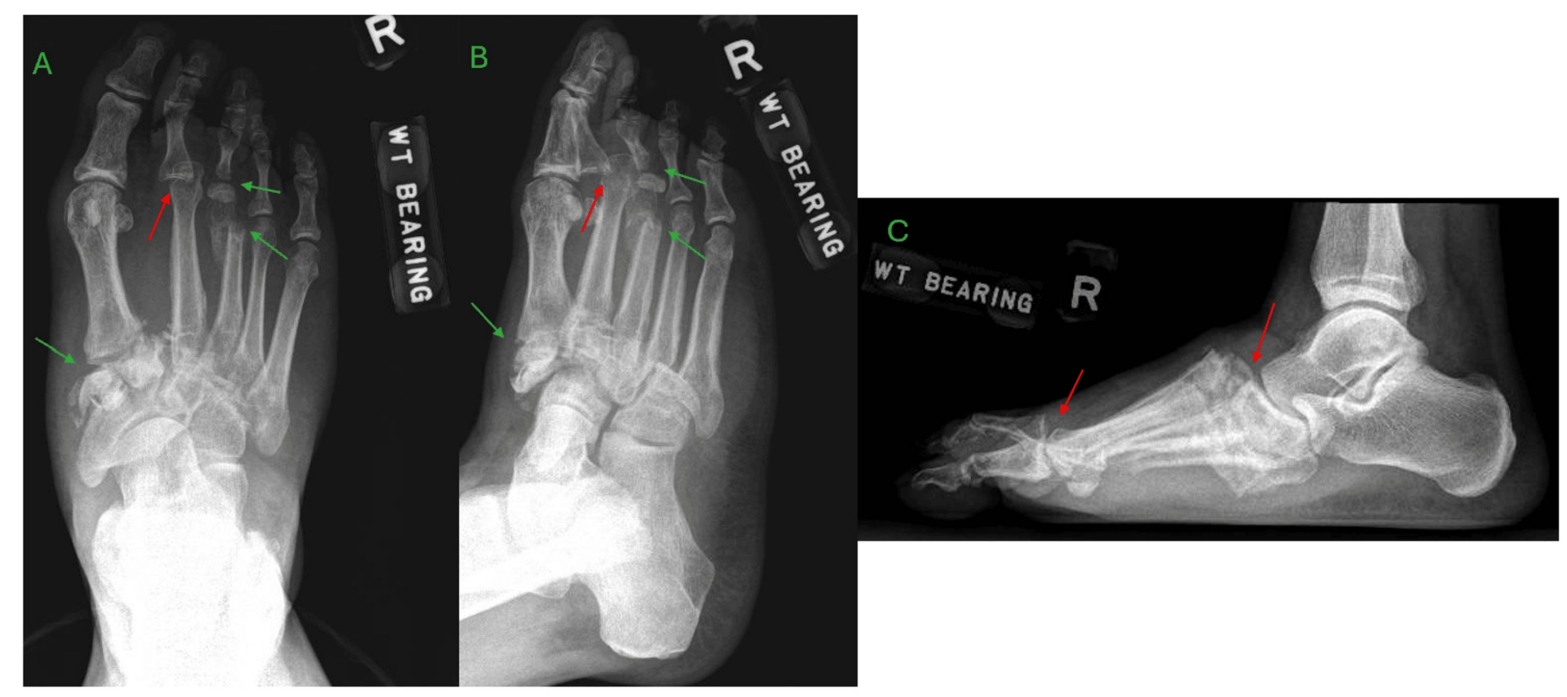
Initial weight-bearing radiographs of the right foot showing features of Charcot arthropathy A – antero-posterior view, B – oblique view, C – lateral view Pertinent features include fractures of the third metatarsal and proximal phalanx, medial cuneiform (green arrows), and dorsomedial subluxation of the second metatarsophalangeal joint and tarsometatarsal joint (red arrows).

**Figure 2:**
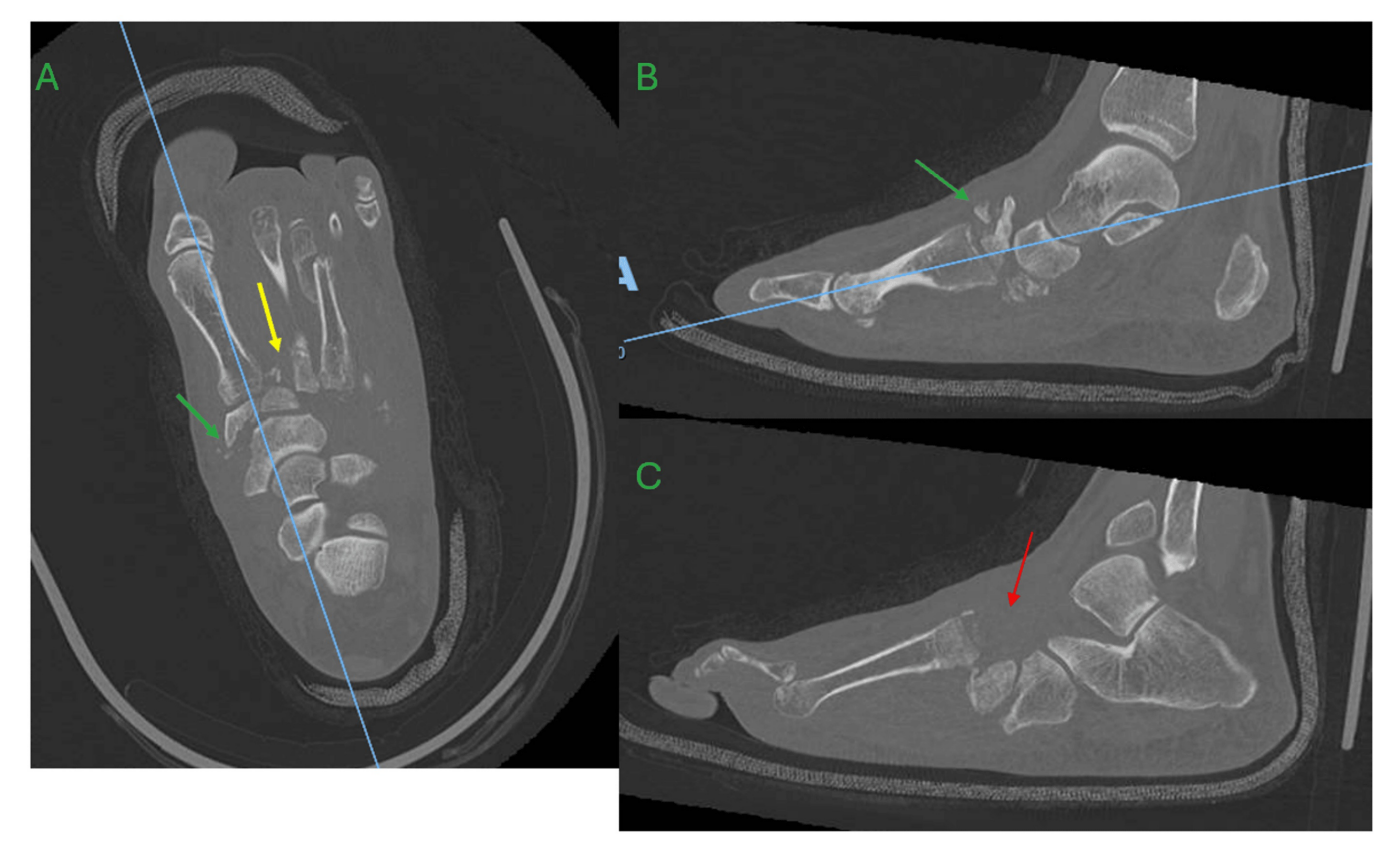
Computer tomography images of the right foot showing features of Charcot arthropathy A – axial cut at the first tarsometatarsal joint level, B – sagittal cut at the first tarsometatarsal joint, C – sagittal cut at the second tarsometatarsal joint Pertinent features include midfoot collapse with tarsometatarsal joint fractures (yellow arrow) and dorsal dislocations (red arrow), as well as comminuted fractures of the cuneiform bones (green arrow).

The most obvious findings on plain radiographs of the foot were displaced fractures of the third metatarsal neck and proximal phalangeal base, as well as dorsomedial subluxation of the second metatarsophalangeal joint (MTPJ). There were also displaced fractures noted in the medial cuneiform (Figure 1). Computer tomography (CT) scans of the foot revealed features of CA, such as midfoot collapse with multiple fractures and dorsal dislocations of the tarsometatarsal joints (TMTJ) from the second to fifth toes, as well as comminuted fractures of the cuneiform bones. No radiological features suggestive of infection were present (Figure 2).

Blood investigations revealed an elevated white blood cell (WBC) count of 11200 µL (reference range: 4500-11000 µL) and a C-reactive protein (CRP) level of 41 mg/L (reference range: <3.0 mg/L). The uric acid level was within normal limits. The patient was referred to the infectious disease service in view of the patient's background of syphilis and the raised inflammatory markers. Various tests, such as blood cultures, were performed, but no bacteria were identified. Hepatitis and human immunodeficiency virus (HIV) screens were negative. The antinuclear antibodies (ANA) test and anti-dsDNA (anti-double-stranded DNA) test were negative, and the serum rheumatoid factor (RF) level was <10. A lumbar puncture was performed. Cerebrospinal fluid (CSF) grew no bacteria, fungal microscopy revealed no organisms, and the acid-fast bacilli (AFB) smear was negative. However, the CSF-VDRL titre was 1:8, and there was a positive LIA syphilis CSF IgG test.

On the basis of these findings, the diagnosis of syphilitic CA was made. The patient was commenced on treatment for tertiary syphilis with a two-week course of intravenous (IV) benzylpenicillin. With respect to CA, the patient required additional time to consider and evaluate the available conservative and surgical management options. Thus, the foot was initially managed with cast immobilization while awaiting completion of the antibiotic regimen for neurosyphilis and the patient’s decision regarding surgery.

**Figure 3:**
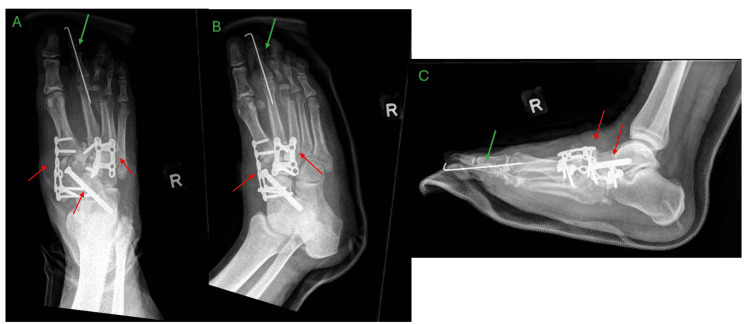
Immediate postoperative radiographs of the right foot showing intact implants after surgery A – anteroposterior view, B – oblique view, C – lateral view, TMTJ – tarsometatarsal joint Deformity correction and fusion of the first tarsometatarsal joint, naviculocuneiform and talonavicular joints, second and third TMTJ fusion (red arrows), as well as second toe deformity correction with Kirschner wiring (green arrow).

**Figure 4:**
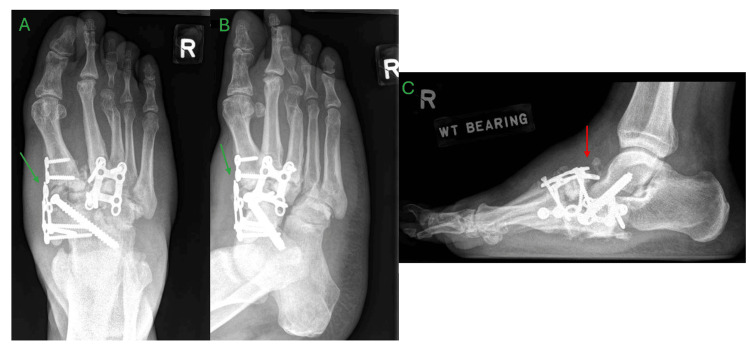
Four-month postoperation radiographs of the right foot showing breakage of the medial plate with progressive midfoot deformity A – anteroposterior view, B – oblique view, C – lateral view Progressive midfoot deformity (red arrow), breakage of the medial plate (green arrows)

Approximately three months after the initial presentation, the patient opted for surgery and underwent exostectomy, deformity correction, fusion of the first tarsometatarsal (TMT) joint, naviculocuneiform and talonavicular joints, fusion of the second and third TMT joints, and second-toe deformity correction with Kirschner wire fixation (Figure 3). At the time of surgery, beaming screws and implants specific for CA were not available in the country. The Kirschner wire was removed at two months post-operation. Although the patient was instructed to maintain a non-weight-bearing status to facilitate adequate fusion, the patient began weight-bearing on the operated limb within two weeks of surgery. This resulted in progressive midfoot arch collapse and worsening rocker-bottom deformity by four months postoperatively, along with failure of the medial plate (Figure 4). However, the patient decided for conservative management.

**Figure 5:**
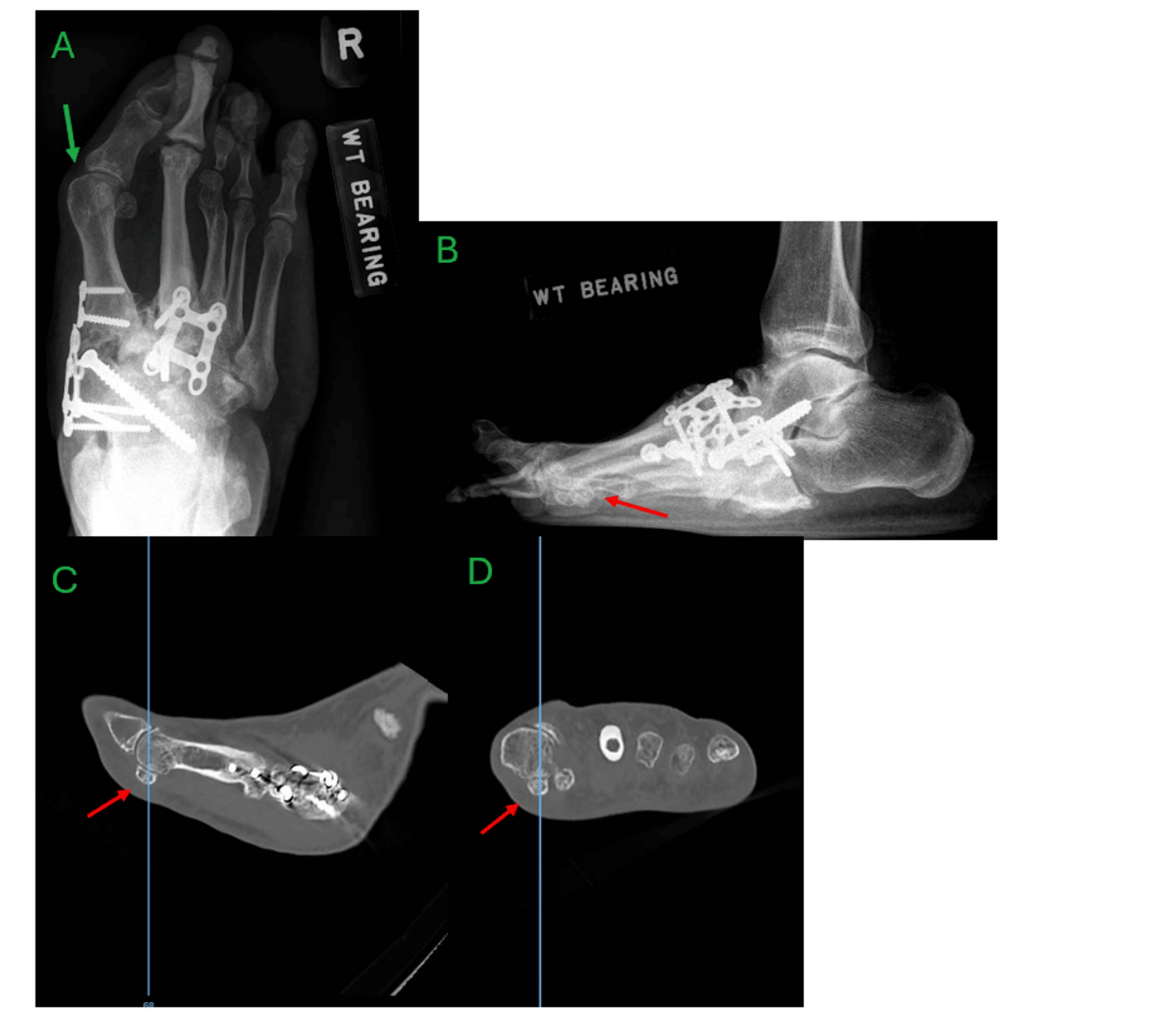
Right foot weight-bearing radiographs and computer tomography showing hallux valgus deformity with plantar osteophytes A – anteroposterior view, B – lateral view, C – sagittal cut at the first metatarsal head, D – axial cut at the first metatarsal head Worsening hallux valgus deformity (green arrow) with plantar osteophytes (red arrows)

**Figure 6:**
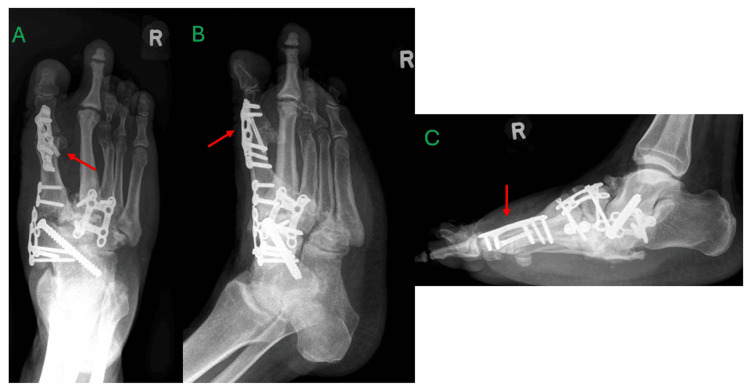
Immediate postoperative radiographs of the right foot after first metatarsophalangeal joint arthrodesis and excision of plantar osteophytes A – anteroposterior view, B – oblique view, C – lateral view First metatarsophalangeal joint arthrodesis (red arrows)

**Figure 7:**
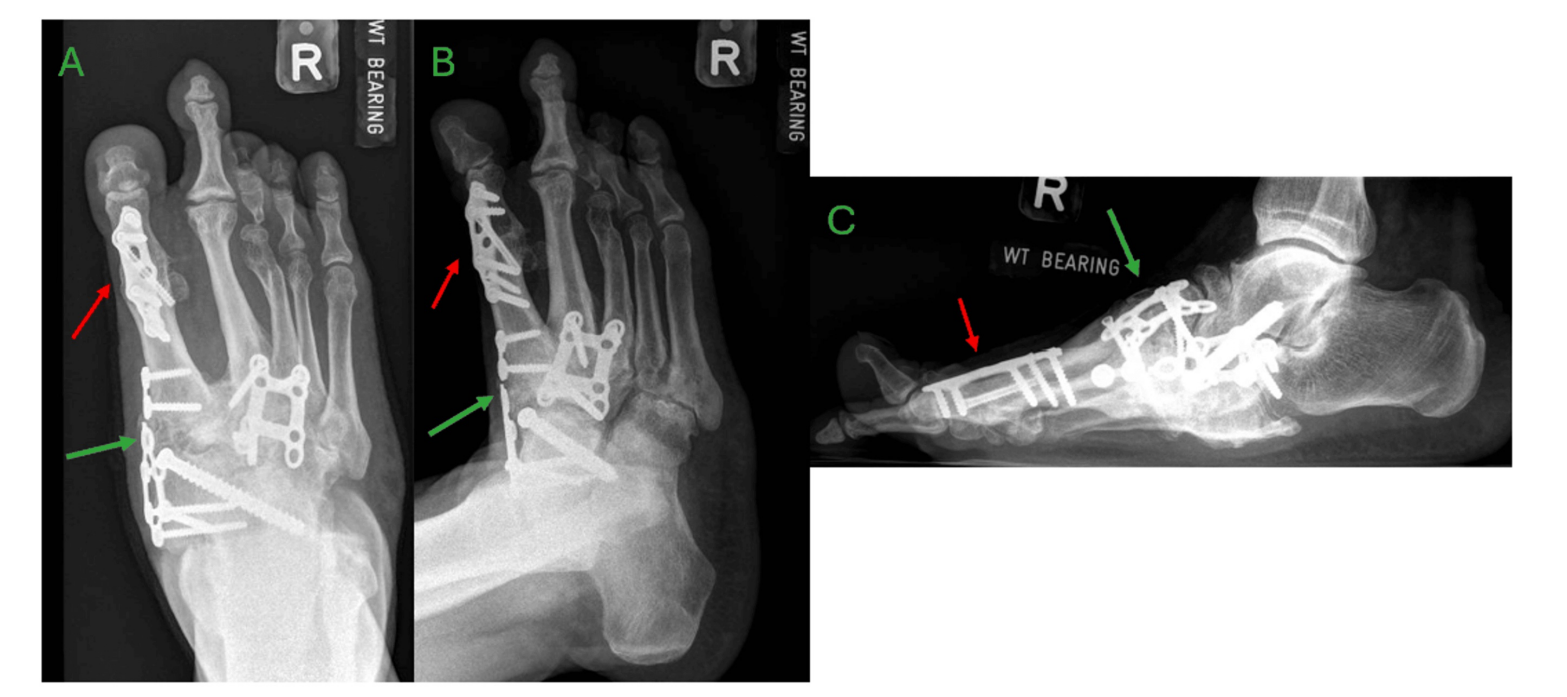
Two years post hallux-valgus correction and osteophyte excision and four years since the first operation, weight-bearing radiographs of the right foot showing no recurrence of hallux valgus deformity and stable midfoot arthropathy A – anteroposterior view, B – oblique view, C – lateral view No recurrence of hallux valgus (red arrows), stable midfoot arthropathy (green arrows)

**Figure 8:**
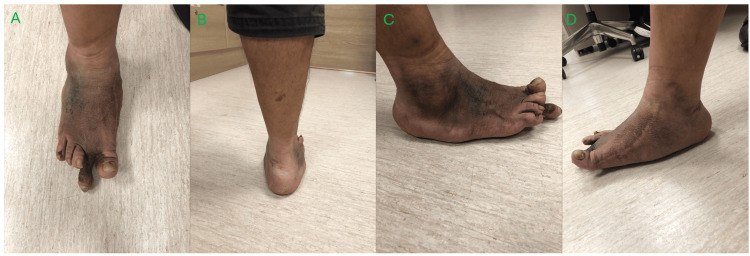
Clinical appearance of the right foot two years post hallux valgus correction and osteophyte excision and four years since the first operation A – anterior view, B – posterior view, C – lateral view, D – medial view

Approximately two years postoperatively, the patient re-presented with the issue of progressive pain over the sole of the first MTPJ. The patient had a hallux valgus deformity with an overriding second toe. Radiographs and a CT scan were performed, showing features of CA (multiple old fractures in the midfoot) and worsening hallux valgus deformity with plantar osteophyte. Besides the known breakage in the medial plate, there were no new implant issues seen, and midfoot arthropathy appeared stable (Figure 5). The patient underwent the first MTPJ arthrodesis and excision of plantar osteophytes in 2021 (Figure 6). Postoperatively, the patient was kept on non-weight-bearing status for two months, after which progressive weight-bearing was initiated and advanced to full weight-bearing. At two years postoperation (which is four years after the first operation), radiographs showed no implant breakage or reoccurrence of hallux valgus deformity, and the midfoot arthropathy remained stable (Figure 7). The patient was able to walk without the use of an aid, and all surgical scars had fully healed with no wound complications (Figure 8).

## Discussion

In the absence of treatment for syphilis infection, neurosyphilis can develop and result in complications such as CA [1]. The current prevalence of syphilitic CA is unknown. Data from the earlier half of the 20th century suggest that it ranges from 1-10% of tabes dorsalis patients [18,19]. CA is characterized by progressive bony destruction with impaired nociceptive and proprioceptive innervation of the affected joints [2]. The exact pathophysiology underlying the destructive process of Charcot arthropathy remains unclear, but likely involves a combination of metabolic, neurologic, and mechanical factors [18,20]. Syphilis has re-emerged in regions such as Europe, North America, and China mainly due to travel and high-risk sexual activity [5,6]. There are various causes of CA, such as syringomyelia, leprosy, alcoholism, and demyelinating peripheral neuropathies [2]. More recently, CA, as a result of medical treatment, such as chemotherapy, has also been seen [21]. However, in today’s context, diabetes remains the most common cause [7]. In recent years, there has been a relative paucity of literature on syphilitic Charcot joints, with most articles reporting on the involvement of hip and knee joints [8-17]. A list of recent syphilitic CA cases and the joints involved has been provided (Table 1).

**Table 1:** Summary of recent cases of syphilitic Charcot arthropathies

Author	Year Published	Age (Years)	Joint Involved	Treatment Summary
Wang et al. [8]	2019	43	Ankle	- Penicillin
Singh et al. [9]	2023	57	Elbow	- Penicillin > Planning for elbow replacement
Drago et al. [10]	2011	69	Hip	- Physiotherapy
Viens et al. [11]	2010	73	Hip	- Penicillin > Physiotherapy
Kuoame et al. [12]	2023	68/54	Knee/Hip	- Penicillin > Physiotherapy
Cardile et al. [13]	2020	67	Knee	- Penicillin > Megaprosthesis > Amputation due to complications (traumatic periprosthetic fracture, infection)
Figueiredo et al. [14]	2018	55	Knee	- Arthrodesis
Labied et al. [15]	2024	59	Knee	- Penicillin > Arthroplasty
Sim et al. [16]	2021	52	Knee	- Amputation (due to unhealthy tissue and wound coverage concerns)
Moudoud et al. [17]	2025	51/61	Knee/Knee	- Penicillin > Physiotherapy

As diabetic CA closely resembles tabetic CA in its clinical presentation, syphilis may be overlooked as a potential underlying aetiology. The principal pitfall in failing to recognise this diagnosis is the assumption that tertiary syphilis no longer occurs in the modern era, leading to a low index of suspicion and, consequently, omission of appropriate investigations. This is particularly relevant in diabetic patients, as diabetes is currently the most common aetiology of foot and ankle CA [7]. This case report highlights the importance of considering syphilitic CA as a differential diagnosis in patients presenting with joint destruction consistent with neuropathic arthropathy involving the foot, even in the modern era. By early consideration and confirmation of the diagnosis, important interventions to treat the underlying cause of CA can be performed. Literature, such as the World Health Organisation (WHO) guidelines, emphasizes the importance of antibiotics in the treatment of all stages of syphilis, including tertiary syphilis [22].

Antibiotic therapy may be considered in accordance with local guidelines for the treatment of tertiary syphilis, following discussion with infectious disease specialists. While antibiotic therapy may not reverse CA, it may contribute to the prevention of further development of symptoms and complications from untreated tertiary syphilis. In this case, the orthopaedic team’s suspicion of syphilis as the underlying cause of CA prompted involvement of infectious disease specialists, who recommended initiating antibiotic therapy. This case report does not imply that all patients with CA of the foot require routine investigation for syphilis. As previously noted, CA may arise from a variety of aetiologies which should also be considered [2,21]. Clinicians should therefore adopt a holistic approach, taking into account the patient’s specific clinical presentation and tailoring investigations accordingly.

Various treatment approaches have been described in the literature. In some cases, antibiotic therapy was not reported, and both conservative and surgical management strategies have been used. When managing a patient with syphilitic CA, we recommend involving infectious disease specialists and treating tertiary syphilis in accordance with local guidelines and policies, while the CA of the foot may be addressed through deformity-correction surgery. 

With regard to the complicated postoperative course in this patient, the adverse outcomes were likely attributable to a combination of patient-related factors and disease complexity, with non-compliance being the principal contributor. The patient failed to adhere to postoperative non-weight-bearing instructions necessary to facilitate adequate fusion. Consequently, the deformity progressed, and hardware failure ensued. This underscores the importance of adherence to prescribed weight-bearing and physiotherapy protocols following major deformity correction procedures, such as those performed for CA.

## Conclusions

Syphilitic CA is a serious complication of neurosyphilis and should be considered in the differential diagnosis of patients presenting with features of neuropathic arthropathy, even in the modern era. This is especially pertinent for cases involving the foot, where diabetes represents the predominant cause, and the resulting similarity in clinical presentation may lead clinicians to erroneously assume a diagnosis of diabetic CA rather than syphilitic CA. Early consideration and confirmation of syphilitic CA enables timely initiation of antibiotic therapy for tertiary syphilis, while the CA can be managed through deformity-correction surgery if required. Furthermore, patient compliance with weight-bearing restrictions after major deformity correction surgery is essential for the patient's outcomes.
